# Robust Consensus of Networked Evolutionary Games with Attackers and Forbidden Profiles †

**DOI:** 10.3390/e20010015

**Published:** 2017-12-29

**Authors:** Yalu Li, Xueying Ding, Haitao Li

**Affiliations:** School of Mathematics and Statistics, Shandong Normal University, Jinan 250014, China

**Keywords:** networked evolutionary game, robust consensus, attackers, forbidden profiles, semi-tensor product of matrices

## Abstract

Using the algebraic state space representation, this paper studies the robust consensus of networked evolutionary games (NEGs) with attackers and forbidden profiles. Firstly, an algebraic form is established for NEGs with attackers and forbidden profiles. Secondly, based on the algebraic form, a necessary and sufficient condition is presented for the robust constrained reachability of NEGs. Thirdly, a series of robust reachable sets is constructed by using the robust constrained reachability, based on which a constructive procedure is proposed to design state feedback controls for the robust consensus of NEGs with attackers and forbidden profiles. Finally, an illustrative example is given to show that the main results are effective.

## 1. Introduction

Since J. M. Smith and G. R. Price’s pioneering works on evolutionary game theory (EGT) [1], the study of EGT has attracted many scholars’ research interests from biology, economics, social science, and so on. It has become a powerful tool to investigate various phenomena raised in social physics, economics and system science [2,3,4]. Evolutionary game played over a network is called a networked evolutionary game (NEG) [4], which mainly studies the influence of the network topology on the evolution of the game, and has been extensively investigated by biologists, physicists, economists and cyberneticists in the last two decades [5,6,7,8,9]. Among this literature, the evolution of cooperation is an important issue [10,11]. In addition, EGT over multilayer networks was also studied [12].

An NEG is mainly made up of three factors, that is, fundamental networked game (FNG), network graph and strategy updating rule (SUR). Among these factors, SUR is the most important one that determines the dynamics of the NEG. Some commonly used SURs include “Myopic Best Response Adjustment”, “Unconditional Imitation”, “Fermi Rule”, “Moran Rule”, and so on. For some comprehensive studies on NEGs, please refer to [13,14].

As an important issue in the study of NEGs, the strategy consensus problem plays an important role in studying the convergence of NEGs to a Nash equilibrium, which can describe the dynamic behaviour of NEGs and provide us a theoretical framework to explore certain kinds of social problems [15]. It is noted that, in the practical NEGs, there often exist attackers who may perturb the strategy choice of each player [16,17] as well as forbidden profiles [18] that correspond to some illegal (undesirable) strategy profiles. For example, in wireless sensor networks, malicious sensor nodes [19,20] who aim to maximize the damage to the networks while minimizing the chance of being caught by some attackers. A motivating example of forbidden profiles comes from a piece of chess board in Figure 1 of [21], where the authors showed that “the profile C2 → B3 is a forbidden move for the black king by the rules of chess.” Thus, it is meaningful to investigate the strategy consensus problem of NEGs with attackers and forbidden profiles.

Recently, Cheng has presented a new matrix product, which is called the semi-tensor product (STP) of matrices [22]. Using the STP, Cheng and his colleagues developed an algebraic state space representation (ASSR) approach for the analysis and control of finite-valued systems [22,23,24,25]. Particularly, the ASSR approach was successfully applied to logical dynamic systems [26,27,28,29,30,31,32,33,34,35,36,37,38,39,40,41,42] and NEGs [14,43,44,45,46]. Fornasini and Valcher presented some necessary and sufficient conditions for the observability and state observers of Boolean control networks [28]. The robust control problem of logical dynamic systems was considered in [33,34]. In [14], an ASSR framework was established for the modeling, analysis and control of NEGs.

In this paper, we study the robust consensus of NEGs with attackers and forbidden profiles by using the ASSR approach. It should be pointed out that our NEG model only considers synchronized strategy updates and average incomes, which is different from NEG models with random sequential strategy updates and total incomes. The main innovation point of this paper are twofold. For one thing, we propose the concept of robust constrained reachability for NEGs, which is effective in dealing with attackers and forbidden profiles. For another, we establish a constructive procedure to design state feedback controls for the robust consensus of NEGs with attackers and forbidden profiles, which is easily operated with the tool of MATLAB (R2016a, The MathWorks, Natick, US State).

In the sequel, the matrix product is the semi-tensor product of matrices, which is defined as: Given two matrices $M\in R^{m\times n}$ and $N\in R^{p\times q}$. Set α=lcm(n,p) be the least common multiple of *n* and *p*. Then,

(1) $$M⋉N=\left(M⊗I_{\frac{\alpha }{n}}\right)\left(N⊗I_{\frac{\alpha }{p}}\right),$$

where ⊗ is the Kronecker product. When n=p, STP is equivalent to the conventional matrix product. Therefore, we omit the symbol “⋉” if no confusion arises in the following. For the detailed properties of STP, please see [22,23]. It is noted that the conventional matrix product requires n=p, while STP is applicable to any two real matrices. Thus, STP is a new matrix product. When considering a finite-valued system such as an NEG, if we identify each strategy as a canonical vector, then one can multiply these canonical vectors by STP (in this case, the conventional matrix product is not valid). In this way, one can convert the dynamics of an NEG into a linear form, which establishes a bridge between NEGs and classic control theory [14]. This is also the motivation for why we use STP to study the robust consensus of NEGs with attackers and forbidden profiles.

The rest of this paper is organized as follows. Section 2 formulates the problem investigated in this paper. In Section 3, the main results of this note are given. In Section 4, an illustrative example is given to show the obtained new results, which is followed by a brief conclusion in Section 5.

The notations of this paper are standard. N, $Z_{+}$ and R denote the sets of natural numbers, positive integers and real numbers, respectively. $D_{k}:=\left\{1,2,\cdots ,k\right\}$. $\Delta_{k}:=\left\{\delta_{k}^{1},\delta_{k}^{2},\delta_{k}^{k}\right\}$, where $\delta_{k}^{i}$ denotes the *i*-th column of the identity matrix $I_{k}$. An n×t matrix *A* is called a logical matrix, if $A=[\delta_{n}^{j_{1}}\;\delta_{n}^{j_{2}}\;\cdots \;\delta_{n}^{j_{t}}]$, which is briefly expressed as $A=\delta_{n}\left[j_{1}\;j_{2}\;\cdots \;j_{t}\right]$. Denote the set of n×t logical matrices by $L_{n\times t}$. $Blk_{l}\left(M\right)$ denotes the *l*-th n×n block of an n×mn matrix *M*. For $M,N\in R^{n\times r}$, the Khatri–Rao product of *M* and *N* is defined as

$$M*N:=[Col_{1}\left(M\right)⊗Col_{1}\left(N\right),\cdots ,Col_{r}\left(M\right)⊗Col_{r}\left(N\right)],$$

where $Col_{s}\left(M\right)$ denotes the *s*-th column of the matrix *M*.

## 2. Problem Formulation

A networked evolutionary game, denoted by ((N,E), *G*, Π), consists of:

- Network graph (N,E), where *N* denotes the set of vertices (players), and *E* denotes the set of edges. Let $N=N_{1}\cup N_{2}\cup N_{3}$ with $N_{i}\cap N_{j}=\emptyset$, ∀i,j=1,2,3,i≠j, where $N_{1}$ is the set of ordinary players, $N_{2}$ is the set of pseudo players who can control the evolutionary game, and $N_{3}$ is the set of attackers (In an NEG, attackers are selfish nodes in the network graph who use the network but do not cooperate. Attacker is different from stochastic player in NEGs with “Fermi rule", where stochastic player is also an ordinary player who may cooperate.) who may destroy the evolutionary game. Set $|N_{1}|=n$, $|N_{2}|=m$ and $|N_{3}|=q$.
- Fundamental networked game, *G*, such that if (i,j)∈E, then *i* and *j* play the FNG repetitively with the strategy set *S*. Without loss of generality, for |S|=k, we let $S=D_{k}$. Denote the strategies of each player in $N_{1}$, $N_{2}$ and $N_{3}$ at time *t* by $z_{i}\left(t\right)\in D_{k}$, $w_{j}\left(t\right)\in D_{k}$ and $\xi_{l}\left(t\right)\in D_{k}$, respectively, where i=1,⋯,n, j=1,⋯,m and l=1,⋯,q.
- Strategy updating rule, Π. Denote the λ-th step neighborhood of each player $P_{i}\in N$ by $U_{\lambda }\left(i\right)$. When λ=1, we briefly denote by U(i) the one step neighborhood of $P_{i}$. At each time instance, each player $P_{i}$ plays the FNG with its neighbors in U(i), and its average payoff, denoted by $c_{i}$, has the following form:
  (2) $$c_{i}\left(P_{i},P_{j}|j\in U\left(i\right)\right)=\frac{\sum_{j\in U(i)\backslash \{i\}}c_{ij}\left(P_{i},P_{j}\right)}{|U(i)|-1},$$
  where $c_{ij}:S\times S\to R$ denotes the payoff of $P_{i}$ playing with its neighbor $P_{j},j\in U\left(i\right)$. Throughout this paper, the strategy updating rule is described by the following fundamental evolutionary equation:
  (3) $$\begin{array}{ccc} P_{i}\left(t+1\right) & = & f_{i}(\{P_{j}\left(t\right),c_{j}\left(P_{j}\left(t\right),P_{k}\left(t\right)|k\in U\left(j\right)\right)|\\ & & j\in U\left(i\right)\}),t\in N,i\in N_{1}, \end{array}$$
  where $f_{i}$ is determined by the following SUR (Unconditional Imitation with Fixed Priority): $P_{i}\left(t+1\right)$ is selected as the best strategy from strategies of its neighbors in U(i) at time *t*. Precisely, if $j^{*}=\arg\max_{j\in U(i)}c_{j}\left(P_{j},P_{k}|k\in U\left(j\right)\right)$, then $P_{i}\left(t+1\right)=P_{j^{*}}\left(t\right)$. When the neighbors with maximum payoff are not unique, say, $\arg\max_{j\in U(i)}c_{j}\left(P_{j},P_{k}|k\in U\left(j\right)\right):=\left\{j_{1}^{*},\cdots ,j_{r}^{*}\right\}$, we choose $j^{*}=\min\left\{j_{1}^{*},\cdots ,j_{r}^{*}\right\}$.

We give an example to demonstrate how to use the SUR to determine the fundamental evolutionary equation.

**Figure 1:**
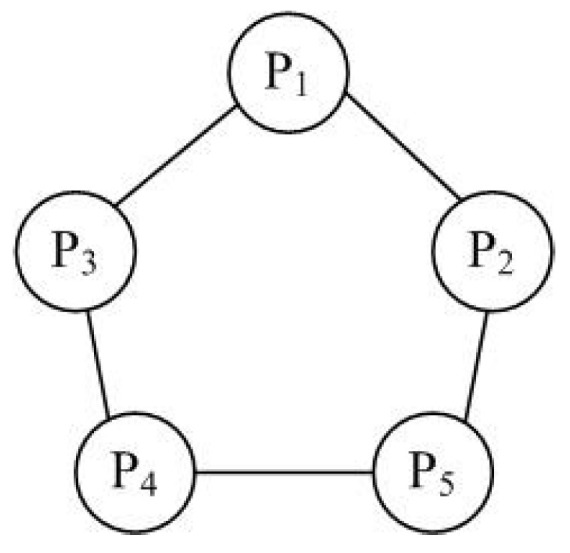
Network graph of the NEG, where $P_{1}$, $P_{2}$ and $P_{3}$ denote ordinary players, while $P_{4}$ and $P_{5}$ are control and attacker, respectively.

**Example** **1.** *Consider an NEG consisting of five players, in which the set of players is denoted by $N=\{P_{1},P_{2},P_{3},P_{4},P_{5}\}$, and the network graph of the game is shown in Figure 1. The basic game of this NEG is the snowdrift game [14], whose payoff matrix is given in Table 1, where “cooperate” and “defect” are denoted by “*1*” and “*2*”, respectively. Hence, all the players have the same strategy set S={1,2}. In this NEG, $P_{4}$ is assumed to be a pseudo player who can freely choose its own strategy at each step, while $P_{5}$ an attacker who may destroy the evolutionary game. Denote the strategies of $P_{1}$, $P_{2}$, $P_{3}$, $P_{4}$ and $P_{5}$ at time t by $P_{1}\left(t\right)$, $P_{2}\left(t\right)$, $P_{3}\left(t\right)$, $P_{4}\left(t\right)$ and $P_{5}\left(t\right)$, respectively.*

Using the SUR of this paper, $f_{1}$, $f_{2}$ and $f_{3}$ can be figured out as in Table 2. From Table 2, one can clearly see the changes of each player’s payoff and strategy with the changes of its neighbors. However, it is not easy to analyze the evolution of the NEG according to Table 2. Thus, we need to establish a mathematical expression of the NEG based on Table 2.

Let “$\delta_{2}^{1}$” be the vector form of strategy “1” and “$\delta_{2}^{2}$” be the vector form of strategy “2”. Denote $z_{1}\left(t\right)=P_{1}\left(t\right)$, $z_{2}\left(t\right)=P_{2}\left(t\right)$, $z_{3}\left(t\right)=P_{3}\left(t\right)$, $w\left(t\right)=P_{4}\left(t\right)$, and $\xi \left(t\right)=P_{5}\left(t\right)$. According to Table 2, we can obtain the structural matrix of each $f_{\kappa }$ as follows:

(4) $$\begin{array}{ccc} z_{\kappa }\left(t+1\right) & = & f_{\kappa }\left(z_{1}\left(t\right),z_{2}\left(t\right),z_{3}\left(t\right),w\left(t\right),\xi \left(t\right)\right)\\ & = & M_{\kappa }z_{1}\left(t\right)z_{2}\left(t\right)z_{3}\left(t\right)w\left(t\right)\xi \left(t\right),\kappa =1,2,3, \end{array}$$

where

$$\begin{array}{ccc} M_{1} & = & \delta_{2}[1\;2\;2\;2\;2\;1\;1\;2\;1\;1\;2\;2\;2\;1\;2\;2\\ & & \;1\;2\;1\;2\;2\;2\;1\;2\;1\;1\;1\;1\;2\;2\;2\;2],\\ M_{2} & = & \delta_{2}[1\;1\;2\;2\;2\;1\;1\;1\;1\;1\;2\;2\;2\;1\;2\;2\\ & & \;2\;2\;1\;2\;2\;2\;2\;2\;1\;1\;1\;2\;2\;1\;2\;2],\\ M_{3} & = & \delta_{2}[1\;2\;1\;2\;2\;1\;1\;1\;2\;1\;2\;2\;2\;2\;2\;1\\ & & \;1\;2\;1\;2\;2\;2\;1\;2\;1\;1\;1\;2\;2\;2\;1\;2]. \end{array}$$

Noting that each column of $M_{\kappa }$ corresponds to a prescribed value of $f_{\kappa }$ in Table 2.

In the following, motivated by Example 1, we establish the algebraic form of NEGs with attackers.

Identify each strategy $\lambda \in S=D_{k}$ as the canonical vector form $\delta_{k}^{\lambda }$, λ=1,2,⋯,k. Let $\xi \left(t\right)=\xi_{1}\left(t\right)⋉\cdots ⋉\xi_{q}\left(t\right)$, $w\left(t\right)=w_{1}\left(t\right)⋉\cdots ⋉w_{m}\left(t\right)$ and $z\left(t\right)=z_{1}\left(t\right)⋉\cdots ⋉z_{n}\left(t\right)$. For each evolutionary dynamic equation (3), one can draw a table like Table 2. From the table, one can find a matrix $M_{i}\in L_{k\times k^{m+n+q}}$ such that

(5) $$z_{i}\left(t+1\right)=M_{i}\xi \left(t\right)w\left(t\right)z\left(t\right),i=1,2,\cdots ,n,$$

where $M_{i}$ is called the structural matrix of $f_{i}$.

Multiplying all the *n* Equations in (5) together, we obtain the algebraic form of NEGs with attackers as follows:

(6) z(t+1)=Lξ(t)w(t)z(t),

where $L=M_{1}*M_{2}*\cdots *M_{n}\in L_{k^{n}\times k^{n+m+q}}$.

In this paper, we assume that the set of strategy profiles in $N_{1}$ takes values from the following forbidden profiles set (In an NEG, forbidden profiles set is a set strategy profiles which are illegal according to rules, laws and regulations of the game.):

(7) $$C_{z}=\left\{\delta_{k^{n}}^{i_{l}}:l=1,2,\cdots ,r\right\},$$

where $1\leq i_{1}<i_{2}<\cdots <i_{r}\leq k^{n}$ and $|C_{z}|=r$.

Now, we introduce the robust consensus problem studied in this paper.

**Definition** **1.** *Consider the NEG (6) with attackers and forbidden profiles set $C_{z}$. Let $\eta \in \Delta_{k}$ and $\eta^{n}\in C_{z}$ be given. The NEG is said to achieve robust consensus at $\eta \in \Delta_{k}$, if there exist a positive integer τ and a control sequence {w(t):t∈N} such that*

(i) $z\left(t;z\left(0\right),w\left(t\right),\xi \left(t\right)\right)\in C_{z}$ holds for ∀t∈N, $\forall \;z\left(0\right)\in C_{z}$ and $\forall \;\xi \left(t\right)\in \Delta_{k^{q}}$;
(ii) $z\left(t;z\left(0\right),w\left(t\right),\xi \left(t\right)\right)=\eta^{n}$ holds for ∀t≥τ, $\forall \;z\left(0\right)\in C_{z}$ and $\forall \;\xi \left(t\right)\in \Delta_{k^{q}}$.

We aim to design a state feedback control in the form of

(8) $$\begin{array}{c} \left\{\begin{array}{c} w_{1}\left(t\right)=b_{1}\left(z_{1}\left(t\right),\cdots ,z_{n}\left(t\right)\right),\\ \;\;\;\;\vdots \\ w_{m}\left(t\right)=b_{m}\left(z_{1}\left(t\right),\cdots ,z_{n}\left(t\right)\right), \end{array}\right. \end{array}$$

where $b_{i}:D_{k}^{n}\to D_{k}$ are *k*-valued logical functions, which needs to be determined, under which the NEG (6) with attackers and forbidden profiles set $C_{z}$ achieves robust consensus at $\eta \in \Delta_{k}$.

Assume that the structural matrix of $b_{i}$ is $B_{i}$, i=1,⋯,m. Then, by using the Khatri–Rao product of matrices, the state feedback control (8) can be described in the following form:

(9) w(t)=Bz(t),

where $B=B_{1}*B_{2}*\cdots *B_{m}\in L_{k^{m}\times k^{n}}$ is called the state feedback gain matrix. Thus, our objective becomes how to design the state feedback gain matrix $B\in L_{k^{m}\times k^{n}}$ such that the robust consensus achieves.

## 3. Main Results

In this section, we firstly present a necessary and sufficient condition for the robust constrained reachability of NEGs with attackers and forbidden profiles, based on which we propose a constructive procedure to design the state feedback gain matrix *B* for the robust consensus of NEGs with attackers and forbidden profiles.

Firstly, we give the definition for the robust constrained reachability of NEGs with attackers and forbidden profiles, which is crucial to the robust consensus of NEGs.

**Definition** **2.** *Consider the NEG (6) with attackers and forbidden profiles.*

(i) $z_{d}\in C_{z}$ is said to be one step robustly reachable from $z_{0}\in C_{z}$, if there exists a control $w\in \Delta_{k^{m}}$ such that $z_{d}=L⋉\xi ⋉w⋉z_{0}$ holds for any $\xi \in \Delta_{k^{q}}$.
(ii) A nonempty set $\Omega \subseteq C_{z}$ is said to be one step robustly reachable from $z_{0}\in C_{z}$, if there exist a control $w\in \Delta_{k^{m}}$ and $z_{\xi }\in \Omega$ (depending on ξ) such that $z_{\xi }=L⋉\xi ⋉w⋉z_{0}$ holds for any $\xi \in \Delta_{k^{q}}$.

In the following, we present a criterion for the robust constrained reachability of NEGs with attackers and forbidden profiles.

Consider the NEG (6). Split $L\in L_{k^{n}\times k^{m+n+q}}$ into $k^{q}$ blocks as

(10) $$L=[L_{1}\;L_{2}\;\cdots \;L_{k^{q}}],$$

where $L_{s}\in L_{k^{n}\times k^{m+n}},s=1,2,\cdots ,k^{q}$. Split each $L_{s}$ into $k^{m}$ blocks as

(11) $$L_{s}=\left[L_{s}^{1}\;L_{s}^{2}\;\cdots \;L_{s}^{k^{m}}\right],$$

where $L_{s}^{j}\in L_{k^{n}\times k^{n}},j=1,2,\cdots ,k^{m}$.

Define

(12) $$\hat{L}=\left[{\hat{L}}_{1}\;{\hat{L}}_{2}\;\cdots \;{\hat{L}}_{k^{q}}\right]\in R^{r\times rk^{m+q}},$$

where ${\hat{L}}_{s}=\left[{\hat{L}}_{s}^{1}\;{\hat{L}}_{s}^{2}\;\cdots \;{\hat{L}}_{s}^{k^{m}}\right]\in R^{r\times rk^{m}}$, and

(13) $${\hat{L}}_{s}^{j}={\left(\delta_{k^{n}}\left[i_{1}\;i_{2}\;\cdots \;i_{r}\right]\right)}^{T}L_{s}^{j}\left(\delta_{k^{n}}\left[i_{1}\;i_{2}\;\cdots \;i_{r}\right]\right)\in R^{r\times r}.$$

Obviously, ${\hat{L}}_{s}^{j}$ is obtained from $L_{s}^{j}$ by deleting all the elements in the rows and columns with indexes $\left\{1,2,\cdots ,k^{n}\right\}\backslash \left\{i_{1},i_{2},\cdots ,i_{r}\right\}$.

**Lemma** **1.** If $\delta_{k^{n}}^{i_{\alpha }}=L_{s}^{j}\delta_{k^{n}}^{i_{\beta }}$, α,β∈{1,2,⋯,r}, then $\delta_{r}^{\alpha }={\hat{L}}_{s}^{j}\delta_{r}^{\beta }$.

**Proof.**  On one hand, it is easy to see from $\delta_{k^{n}}^{i_{\alpha }}=L_{s}^{j}\delta_{k^{n}}^{i_{\beta }}$ that ${\left(\delta_{k^{n}}\left[i_{1}\;i_{2}\;\cdots \;i_{r}\right]\right)}^{T}\delta_{k^{n}}^{i_{\alpha }}={\left(\delta_{k^{n}}\left[i_{1}\;i_{2}\;\cdots \;i_{r}\right]\right)}^{T}L_{s}^{j}\delta_{k^{n}}^{i_{\beta }}$. On the other hand, a simple calculation shows that

$${\left(\delta_{k^{n}}\left[i_{1}\;i_{2}\;\cdots \;i_{r}\right]\right)}^{T}\delta_{k^{n}}^{i_{\alpha }}=\delta_{r}^{\alpha }$$

and

$$\delta_{k^{n}}^{i_{\beta }}=\delta_{k^{n}}\left[i_{1}\;i_{2}\;\cdots \;i_{r}\right]\delta_{r}^{\beta }.$$

Therefore, $\delta_{r}^{\alpha }={\hat{L}}_{s}^{j}\delta_{r}^{\beta }$. This completes the proof. ☐

Based on Definition 2 and Lemma 1, we have the following result on the robust constrained reachability of NEGs with attackers and forbidden profiles.

**Theorem** **1.** *Consider the NEG (6) with attackers and forbidden profiles set $C_{z}$.*

(i) *$z_{d}=\delta_{k^{n}}^{i_{\alpha }}\in C_{z}$ is one step robustly reachable from $z_{0}=\delta_{k^{n}}^{i_{\beta }}\in C_{z}$, if and only if there exists a positive integer $1\leq j\leq k^{m}$ such that*
  (14) $$\sum_{s=1}^{k^{q}}{\left({\hat{L}}_{s}^{j}\right)}_{\alpha ,\beta }=k^{q}.$$
(ii) *A nonempty set $\Omega \subseteq C_{z}$ is one step robustly reachable from $z_{0}=\delta_{k^{n}}^{i_{\beta }}\in C_{z}$, if and only if there exists a positive integer $1\leq j\leq k^{m}$ such that*
  (15) $$\sum_{s=1}^{k^{q}}\sum_{\delta_{k^{n}}^{i_{\alpha }}\in \Omega }{\left({\hat{L}}_{s}^{j}\right)}_{\alpha ,\beta }=k^{q}.$$

**Proof.**  We firstly prove conclusion (i).

(Necessity) Suppose that $z_{d}=\delta_{k^{n}}^{i_{\alpha }}\in C_{z}$ is one step robustly reachable from $z_{0}=\delta_{k^{n}}^{i_{\beta }}\in C_{z}$. Then, there exists a control $w=\delta_{k^{m}}^{j}$ such that $\delta_{k^{n}}^{i_{\alpha }}=L⋉\delta_{k^{q}}^{s}⋉\delta_{k^{m}}^{j}⋉\delta_{k^{n}}^{i_{\beta }}$ holds for any $s=1,2,\cdots ,k^{q}$. By Lemma 1, one can see that $\delta_{r}^{\alpha }={\hat{L}}_{s}^{j}\delta_{r}^{\beta }$ holds for any $s=1,2,\cdots ,k^{q}$. Thus, ${\left({\hat{L}}_{s}^{j}\right)}_{\alpha ,\beta }=1$ holds for any $s=1,2,\cdots ,k^{q}$, which implies that (14) holds.

(Sufficiency) Assuming that (14) holds for some integer $1\leq j\leq k^{m}$, that is, ${\left({\hat{L}}_{s}^{j}\right)}_{\alpha ,\beta }=1$ holds for any $s=1,2,\cdots ,k^{q}$, which implies that $\delta_{r}^{\alpha }={\hat{L}}_{s}^{j}\delta_{r}^{\beta }$ holds for any $s=1,2,\cdots ,k^{q}$. Thus, $\left(\delta_{k^{n}}\left[i_{1}\;i_{2}\;\cdots \;i_{r}\right]\right)\delta_{r}^{\alpha }=\left(\delta_{k^{n}}\left[i_{1}\;i_{2}\;\cdots \;i_{r}\right]\right){\hat{L}}_{s}^{j}\delta_{r}^{\beta }$ holds for any $s=1,2,\cdots ,k^{q}$. By the construction of ${\hat{L}}_{s}^{j}$, we can obtain that

$$\begin{array}{c} \left(\delta_{k^{n}}\left[i_{1}\;i_{2}\;\cdots \;i_{r}\right]\right)\delta_{r}^{\alpha }=\Gamma L_{s}^{j}\left(\delta_{k^{n}}\left[i_{1}\;i_{2}\;\cdots \;i_{r}\right]\right)\delta_{r}^{\beta }, \end{array}$$

that is, $\delta_{k^{n}}^{i_{\alpha }}=\Gamma ⋉L_{s}^{j}⋉\delta_{k^{n}}^{i_{\beta }}$ holds for any $s=1,2,\cdots ,k^{q}$, where

(16) $$\Gamma :=\left(\delta_{k^{n}}\left[i_{1}\;i_{2}\;\cdots \;i_{r}\right]\right){\left(\delta_{k^{n}}\left[i_{1}\;i_{2}\;\cdots \;i_{r}\right]\right)}^{T}.$$

Noticing that only $\Gamma ⋉\delta_{k^{n}}^{i_{\alpha }}=\delta_{k^{n}}^{i_{\alpha }}$, one can see that

$$\delta_{k^{n}}^{i_{\alpha }}=L_{s}^{j}⋉\delta_{k^{n}}^{i_{\beta }}=L⋉\delta_{k^{q}}^{s}⋉\delta_{k^{m}}^{j}⋉\delta_{k^{n}}^{i_{\beta }}$$

holds for any $s=1,2,\cdots ,k^{q}$. By Definition 2, $z_{d}=\delta_{k^{n}}^{i_{\alpha }}\in C_{z}$ is one step robustly reachable from $z_{0}=\delta_{k^{n}}^{i_{\beta }}\in C_{z}$.

Next, we prove conclusion (ii).

(Necessity) Assuming that Ω is one step robustly reachable from $z_{0}=\delta_{k^{n}}^{i_{\beta }}\in C_{z}$, then there exist a control $w=\delta_{k^{m}}^{j}$ and $z_{\xi }=\delta_{k^{n}}^{i_{\alpha (\xi )}}\in \Omega$ such that $\delta_{k^{n}}^{i_{\alpha (\xi )}}=L⋉\delta_{k^{q}}^{s}⋉\delta_{k^{m}}^{j}⋉\delta_{k^{n}}^{i_{\beta }}=L_{s}^{j}\delta_{k^{n}}^{i_{\beta }}$ holds for any $\xi =\delta_{k^{q}}^{s}\in \Delta_{k^{q}}$. By Lemma 1, we know that $\delta_{r}^{\alpha (\xi )}={\hat{L}}_{s}^{j}\delta_{r}^{\beta }$ holds for any $s=1,2,\cdots ,k^{q}$. Since $Col_{\beta }\left({\hat{L}}_{s}^{j}\right)$ is a logical vector, one can see that $\sum_{\delta_{k^{n}}^{i_{\alpha }}\in \Omega }{\left({\hat{L}}_{s}^{j}\right)}_{\alpha ,\beta }=1$ holds for any $s=1,2,\cdots ,k^{q}$, which implies that (15) holds.

(Sufficiency) Suppose that (15) holds for some integer $1\leq j\leq k^{m}$. Since $Col_{\beta }\left({\hat{L}}_{s}^{j}\right)\in L_{r\times 1}$, we know that $\sum_{\delta_{k^{n}}^{i_{\alpha }}\in \Omega }{\left({\hat{L}}_{s}^{j}\right)}_{\alpha ,\beta }=1$ holds for $\forall \;s=1,2,\cdots ,k^{q}$. Therefore, for each $\xi =\delta_{k^{q}}^{s}\in \Delta_{k^{q}}$, there exists $\delta_{k^{n}}^{i_{\alpha (\xi )}}\in \Omega$ such that ${\left({\hat{L}}_{s}^{j}\right)}_{\alpha (\xi ),\beta }=1$, which implies that $\delta_{r}^{\alpha (\xi )}={\hat{L}}_{s}^{j}\delta_{r}^{\beta }$ holds for any $\xi =\delta_{k^{q}}^{s}\in \Delta_{k^{q}}$. Thus, $\left(\delta_{k^{n}}\left[i_{1}\;i_{2}\;\cdots \;i_{r}\right]\right)\delta_{r}^{\alpha (\xi )}=\left(\delta_{k^{n}}\left[i_{1}\;i_{2}\;\cdots \;i_{r}\right]\right){\hat{L}}_{s}^{j}\delta_{r}^{\beta }$ holds for any $\xi =\delta_{k^{q}}^{s}\in \Delta_{k^{q}}$. By the construction of ${\hat{L}}_{s}^{j}$, we can obtain that

$$\begin{array}{c} \left(\delta_{k^{n}}\left[i_{1}\;i_{2}\;\cdots \;i_{r}\right]\right)\delta_{r}^{\alpha (\xi )}=\Gamma L_{s}^{j}\left(\delta_{k^{n}}\left[i_{1}\;i_{2}\;\cdots \;i_{r}\right]\right)\delta_{r}^{\beta }, \end{array}$$

that is, $\delta_{k^{n}}^{i_{\alpha (\xi )}}=\Gamma ⋉L_{s}^{j}⋉\delta_{k^{n}}^{i_{\beta }}$ holds for any $\xi =\delta_{k^{q}}^{s}\in \Delta_{k^{q}}$, where Γ is given in (16). It is easy to see from $\Gamma ⋉\delta_{k^{n}}^{i_{\alpha (\xi )}}=\delta_{k^{n}}^{i_{\alpha (\xi )}}$ and $\Gamma ⋉\delta_{k^{n}}^{i_{\rho }}\neq \delta_{k^{n}}^{i_{\alpha (\xi )}}$, ∀ρ≠α(ξ) that

$$\delta_{k^{n}}^{i_{\alpha (\xi )}}=L_{s}^{j}⋉\delta_{k^{n}}^{i_{\beta }}=L⋉\delta_{k^{q}}^{s}⋉\delta_{k^{m}}^{j}⋉\delta_{k^{n}}^{i_{\beta }}$$

holds for any $\xi =\delta_{k^{q}}^{s}\in \Delta_{k^{q}}$. By Definition 2, Ω is one step robustly reachable from $z_{0}=\delta_{k^{n}}^{i_{\beta }}\in C_{z}$. This completes the proof. ☐

Based on the robust constrained reachability of NEGs with attackers and forbidden profiles, we inductively construct a series of robust reachable sets as follows. Let $\eta^{n}=\delta_{k^{n}}^{c}\in C_{z}$, where $\eta \in \Delta_{k}$ and *c* is uniquely determined by η. For example, if $\eta =\delta_{k}^{1}$, then c=1; if $\eta =\delta_{k}^{k}$, then $c=k^{n}$. Define

(17) $$\begin{array}{cc} \Omega_{1}\left(\eta \right)=\{ & \delta_{k^{n}}^{i_{\alpha }}\in C_{z}:\;\mathrm{there}\;\mathrm{exists}\;\mathrm{an}\;\mathrm{integer}\;1\leq j\leq k^{m}\\ & \mathrm{such}\;\mathrm{that}\;\sum_{s=1}^{k^{q}}{\left({\hat{L}}_{s}^{j}\right)}_{c,\alpha }=k^{q}\}, \end{array}$$

(18) $$\begin{array}{c} \Omega_{\gamma }\left(\eta \right)=\{\delta_{k^{n}}^{i_{\alpha }}\in C_{z}:\;\mathrm{there}\;\mathrm{exists}\;\mathrm{an}\;\mathrm{integer}\;1\leq j\leq k^{m}\\ \mathrm{such}\;\mathrm{that}\;\sum_{s=1}^{k^{q}}\sum_{\delta_{k^{n}}^{i_{\alpha^{\prime }}}\in \Omega_{\gamma -1}\left(\eta \right)}{\left({\hat{L}}_{s}^{j}\right)}_{\alpha^{\prime },\alpha }=k^{q}\},\;\gamma \geq 2, \end{array}$$

where $\Omega_{1}\left(\eta \right)$ represents the set of states that can robustly reach $\eta^{n}=\delta_{k^{n}}^{c}$ in one step, and $\Omega_{\gamma }\left(\eta \right)$ is the set of states that can robustly reach $\Omega_{\gamma -1}\left(\eta \right)$ in one step. Then, based on a simple calculation, we have the following results.

**Lemma** **2.** If $\eta^{n}\in \Omega_{1}\left(\eta \right)$, then $\Omega_{\gamma }\left(\eta \right)\subseteq \Omega_{\gamma +1}\left(\eta \right)$ holds for any $\gamma \in Z_{+}$.

**Lemma** **3.** If $\eta^{n}\in \Omega_{1}\left(\eta \right)$ and there exists a positive integer γ such that $\Omega_{\gamma }\left(\eta \right)=\Omega_{\gamma +1}\left(\eta \right)$, then $\Omega_{\chi }\left(\eta \right)=\Omega_{\gamma }\left(\eta \right)$ holds for any integer χ≥γ.

Now, based on Lemmas 2 and 3, we give a sufficient condition for the robust consensus of NEGs with attackers and forbidden profiles.

**Theorem** **2.** *The NEG (6) with attackers and forbidden profiles set $C_{z}$ achieves robust consensus at $\eta \in \Delta_{k}$, if there exists an positive integer 1≤τ≤r such that*

(19) $$\left\{\begin{array}{c} \eta^{n}\in \Omega_{1}\left(\eta \right),\\ \Omega_{\tau }\left(\eta \right)=C_{z}. \end{array}\right.$$

**Proof.**  Assuming that (19) holds, we prove that the NEG (6) with attackers and forbidden profiles set $C_{z}$ achieves robust consensus at $\eta \in \Delta_{k}$ by constructing a state feedback gain matrix.

It is easy to see from $\eta^{n}\in \Omega_{1}\left(\eta \right)$ and Lemma 2 that $\Omega_{\gamma }\left(\eta \right)\subseteq \Omega_{\gamma +1}\left(\eta \right)$ holds for any γ=1,⋯,τ−1. For γ=1,2,⋯,τ, let

$$\Omega_{\gamma }^{\circ }\left(\eta \right)=\Omega_{\gamma }\left(\eta \right)\backslash \Omega_{\gamma -1}\left(\eta \right),$$

where $\Omega_{0}\left(\eta \right):=\emptyset$. Then, for $\forall \;\gamma_{1}\neq \gamma_{2}\in \left\{1,2,\cdots ,\tau \right\}$, $\Omega_{\gamma_{1}}^{\circ }\left(\eta \right)$ and $\Omega_{\gamma_{2}}^{\circ }\left(\eta \right)$ are disjoint sets. In addition, $\Omega_{\tau }\left(\eta \right)=C_{z}$ implies that $\overset{\tau }{\underset{\gamma =1}{⋃}}\Omega_{\gamma }^{\circ }\left(\eta \right)=C_{z}$. Therefore, for any integer 1≤α≤r, there exists a unique integer $1\leq \gamma_{\alpha }\leq \tau$ such that $\delta_{k^{n}}^{i_{\alpha }}\in \Omega_{\gamma_{\alpha }}^{\circ }\left(\eta \right)$. We obtain the following two cases:

(i) When $\gamma_{\alpha }=1$, there exists $1\leq \sigma_{\alpha }\leq k^{m}$ such that
  $$\sum_{s=1}^{k^{q}}{\left({\hat{L}}_{s}^{\sigma_{\alpha }}\right)}_{c,\alpha }=k^{q}.$$
(ii) When $2\leq \gamma_{\alpha }\leq \tau$, there exists $1\leq \sigma_{\alpha }\leq k^{m}$ such that
  $$\sum_{s=1}^{k^{q}}\sum_{\delta_{k^{n}}^{i_{\alpha^{\prime }}}\in \Omega_{\gamma_{\alpha }-1}\left(\eta \right)}{\left({\hat{L}}_{s}^{\sigma_{\alpha }}\right)}_{\alpha^{\prime },\alpha }=k^{q}.$$

Set $B=\delta_{k^{m}}\left[\sigma_{1}\;\sigma_{2}\;\cdots \;\sigma_{k^{n}}\right]\in L_{k^{m}\times k^{n}}$, where

(20) $$\left\{\begin{array}{c} \sigma_{l}=\sigma_{\alpha },\;\mathrm{if}\;l=i_{\alpha },\alpha \in \left\{1,2,\cdots ,r\right\};\\ \sigma_{l}\in \left\{1,2,\cdots ,k^{m}\right\},\;\mathrm{otherwise}. \end{array}\right.$$

Then, under the control w(t)=Bz(t), for any initial state $z\left(0\right)=\delta_{k^{n}}^{i_{\alpha }}\in C_{z}$, it is easy to obtain that $z\left(\gamma_{\alpha };z\left(0\right),w,\xi \right)=\eta^{n}$ holds for any $\left\{\xi \left(t\right):t=0,1,\cdots ,\gamma_{\alpha }-1\right\}\subseteq \Delta_{k^{q}}$ and any integer 1≤α≤r. Since $\eta^{n}\in \Omega_{1}\left(\eta \right)$, we can obtain that $z\left(t;z\left(0\right),w,\xi \right)=\eta^{n}$ holds for ∀t≥τ, $\forall \;z\left(0\right)\in C_{z}$ and $\forall \;\left\{\xi \left(t\right):t\in N\right\}\subseteq \Delta_{k^{q}}$, which implies that the NEG (6) with attackers and forbidden profiles set $C_{z}$ can achieve robust consensus at η under the control $w\left(t\right)=\delta_{k^{m}}\left[\sigma_{1}\;\sigma_{2}\;\cdots \;\sigma_{k^{n}}\right]z\left(t\right)$. This completes the proof. ☐

**Remark** **1.** *Based the proof of Theorem 2, one can design a state feedback control for the robust consensus of NEGs with attackers and forbidden profiles as follows:*

(1) Calculate $\Omega_{\gamma }^{\circ }\left(\eta \right)$, γ=1,2,⋯,τ.
(2) *For any 1≤α≤r which corresponds to a unique integer $1\leq \gamma_{\alpha }\leq \tau$ such that $\delta_{k^{n}}^{i_{\alpha }}\in \Omega_{\gamma_{\alpha }}^{\circ }\left(\eta \right)$, let $1\leq \sigma_{\alpha }\leq k^{m}$ be such that*
  $$\begin{array}{c} \left\{\begin{array}{c} \sum_{s=1}^{k^{q}}{\left({\hat{L}}_{s}^{\sigma_{\alpha }}\right)}_{c,\alpha }=k^{q},\;\gamma_{\alpha }=1;\\ \sum_{s=1}^{k^{q}}\sum_{\delta_{k^{n}}^{i_{\alpha^{\prime }}}\in \Omega_{\gamma_{\alpha }-1}\left(\eta \right)}{\left({\hat{L}}_{s}^{\sigma_{\alpha }}\right)}_{\alpha^{\prime },\alpha }=k^{q},\;2\leq \gamma_{\alpha }\leq \tau . \end{array}\right. \end{array}$$
(3) *A state feedback gain matrix under which the NEG with attackers and forbidden profiles achieves consensus at η can be designed as $B=\delta_{k^{m}}\left[\sigma_{1}\;\sigma_{2}\;\cdots \;\sigma_{k^{n}}\right]$, where*
  (21) $$\left\{\begin{array}{c} \sigma_{l}=\sigma_{\alpha },\;if\;l=i_{\alpha },\alpha \in \left\{1,2,\cdots ,r\right\};\\ \sigma_{l}\in \left\{1,2,\cdots ,k^{m}\right\},\;otherwise. \end{array}\right.$$

Finally, we prove that the condition (19) is also necessary for the robust consensus of NEGs with attackers and forbidden profiles.

**Theorem** **3.** If the NEG (6) with attackers and forbidden profiles set $C_{z}$ achieves robust consensus at $\eta \in \Delta_{k}$, then there exists an integer 1≤τ≤r such that (19) holds.

**Proof.**  Assume that the NEG (6) with attackers and forbidden profiles set $C_{z}$ achieves robust consensus at $\eta \in \Delta_{k}$. Then, one can obtain that

(i) $\eta^{n}$ is one step robustly reachable from itself in one step.
(ii) There exists a positive integer τ such that $\eta^{n}$ is robustly reachable from any $z_{0}\in C_{z}$ at the τ-th step.

By Theorem 1, (17) and (18), conclusion (i) is equivalent to $\eta^{n}\in \Omega_{1}\left(\eta \right)$, and conclusion (ii) is equivalent to $\Omega_{\tau }\left(\eta \right)=C_{z}$. Set τ be the smallest positive integer such that $\Omega_{\tau }\left(\eta \right)=C_{z}$. We prove that τ≤r.

In fact, $\eta^{n}\in \Omega_{1}\left(\eta \right)$ implies that $|\Omega_{1}\left(\eta \right)|\geq 1$. Now, we assume that $|\Omega_{\gamma }\left(\eta \right)|\geq \gamma$ holds for some integer 1≤γ≤τ−1. If $|\Omega_{\gamma +1}\left(\eta \right)|<\gamma +1$, and one can see from $\Omega_{\gamma }\left(\eta \right)\subseteq \Omega_{\gamma +1}\left(\eta \right)$ and $|\Omega_{\gamma }\left(\eta \right)|\geq \gamma$ that $\Omega_{\gamma }\left(\eta \right)=\Omega_{\gamma +1}\left(\eta \right)$. Thus, by Lemma 3, $\Omega_{\gamma }\left(\eta \right)=\Omega_{\tau }\left(\eta \right)=C_{z}$, which is a contradiction to the minimality of τ. Therefore, $|\Omega_{\gamma +1}\left(\eta \right)|\geq \gamma +1$. By induction, $|\Omega_{\gamma }\left(\eta \right)|\geq \gamma$ holds for any integer 1≤γ≤τ. When γ=τ, it is easy to see from $r=|C_{z}\left|=\right|\Omega_{\tau }\left(\eta \right)|\geq \tau$ that τ≤r. This completes the proof. ☐

**Remark** **2.** Theorems 2 and 3 provide a necessary and sufficient condition for the robust consensus of NEGs with attackers and forbidden profiles. Compared with the computer simulation method (which is the main tool to study NEGs in the literature), the STP based theoretical framework avoids the blindness of finding a suitable control strategy. In addition, the STP based main results are easily operated via MATLAB.

## 4. An Illustrative Example

**Figure 2:**
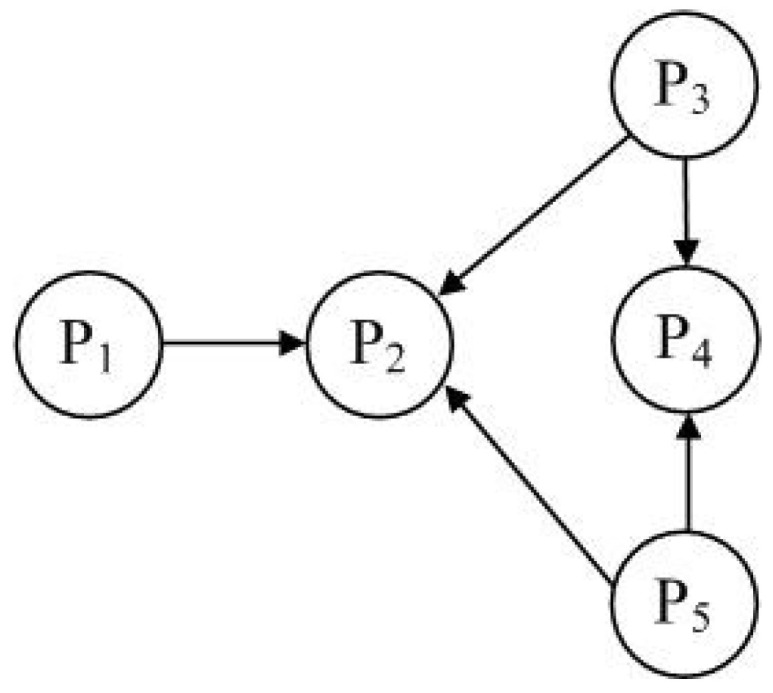
Network graph of the NEG, where $P_{1},P_{3}$ and $P_{5}$ denote small pigs, while $P_{2}$ and $P_{4}$ big pigs. $P_{1}$ and $P_{4}$ are assumed to be attacker and control, respectively.

Consider an NEG consisting of five players, in which the set of players is denoted by $N=\{P_{1},P_{2},P_{3},P_{4},P_{5}\}$ and the network graph of the game is shown in Figure 2. The basic game of this NEG is the Boxed Pigs Game [14], whose payoff matrix is given in Table 3, where “Press” and “Wait” are denoted by “1” and “2”, respectively. Hence, all the players have the same strategy set S={1,2}. In this NEG, $P_{4}$ is assumed to be a control, while $P_{1}$ is assumed to be an attacker. We suppose that $P_{1},P_{3}$ and $P_{5}$ denote small pigs, while $P_{2}$ and $P_{4}$ big pigs. Denote the strategies of $P_{1}$, $P_{2}$, $P_{3}$ ,$P_{4}$ and $P_{5}$ at time *t* by $x_{1}\left(t\right)$, $x_{2}\left(t\right)$, $x_{3}\left(t\right)$, $x_{4}\left(t\right)$ and $x_{5}\left(t\right)$, respectively.

According to the SUR of this paper, we have the following evolutionary dynamic equations:

(22) $$\begin{array}{c} \left\{\begin{array}{c} x_{2}\left(t+1\right)=f_{1}\left(x_{1}\left(t\right),x_{2}\left(t\right),x_{3}\left(t\right),x_{4}\left(t\right),x_{5}\left(t\right)\right),\\ x_{3}\left(t+1\right)=f_{2}\left(x_{1}\left(t\right),x_{2}\left(t\right),x_{3}\left(t\right),x_{4}\left(t\right),x_{5}\left(t\right)\right),\\ x_{5}\left(t+1\right)=f_{3}\left(x_{1}\left(t\right),x_{2}\left(t\right),x_{3}\left(t\right),x_{4}\left(t\right),x_{5}\left(t\right)\right), \end{array}\right. \end{array}$$

where $f_{1},f_{2},f_{3}$ are Boolean functions, which can be uniquely determined by the SUR.

Let “$\delta_{2}^{1}$” be the vector form of “1” and “$\delta_{2}^{2}$” be the vector form of “2”. Using the vector form of $x_{j}\left(t\right),j=1,2,3,4,5$ and letting $z\left(t\right)=x_{2}\left(t\right)⋉x_{3}\left(t\right)⋉x_{5}\left(t\right)$, $w\left(t\right)=x_{4}\left(t\right)$, $\xi \left(t\right)=x_{1}\left(t\right)$, by drawing a table like Table 2, we can obtain the algebraic form of the NEG as follows:

(23) $$\begin{array}{c} \begin{array}{c} z(t+1)=L\xi (t)w(t)z(t), \end{array} \end{array}$$

where

$$\begin{array}{ccc} L & = & \delta_{8}[1\;\;1\;\;1\;\;1\;\;8\;\;8\;\;8\;\;5\;\;4\;\;1\;\;1\;\;1\;\;8\;\;8\;\;8\;\;8\\ & & \;\;\;1\;\;1\;\;1\;\;5\;\;8\;\;5\;\;5\;\;5\;\;4\;\;4\;\;4\;\;5\;\;8\;\;8\;\;8\;\;8]. \end{array}$$

For this example, we assume $C_{z}=\left\{\delta_{8}^{1},\delta_{8}^{3}\right\}$. Our objective is to design a state feedback control in the form of w(t)=Bz(t) (if possible) such that the NEG with attacker $\xi \left(t\right)=x_{1}\left(t\right)$ and forbidden profiles set $C_{z}$ achieves robust consensus at $\eta =\delta_{2}^{1}$.

It is easy to see that

$${\hat{L}}_{1}^{1}=\left[\begin{array}{cc} 1 & 1\\ 0 & 0 \end{array}\right],\;{\hat{L}}_{2}^{1}=\left[\begin{array}{cc} 1 & 1\\ 0 & 0 \end{array}\right],\;{\hat{L}}_{1}^{2}=\left[\begin{array}{cc} 0 & 1\\ 0 & 0 \end{array}\right],\;{\hat{L}}_{2}^{2}=\left[\begin{array}{cc} 0 & 0\\ 0 & 0 \end{array}\right].$$

Hence,

$$\sum_{i=1}^{2}{\left({\hat{L}}_{i}^{1}\right)}_{1,1}=2,\;\sum_{i=1}^{2}{\left({\hat{L}}_{i}^{1}\right)}_{1,2}=2,$$

which together with (17) implies that $\Omega_{1}\left(\eta \right)=C_{z}$. Therefore, by Theorem 2 and Remark 1, under the state feedback gain matrix

$$B=\delta_{2}\left[1\;\sigma_{2}\;1\;\sigma_{4}\;\sigma_{5}\;\sigma_{6}\;\sigma_{7}\;\sigma_{8}\right],$$

where $\sigma_{i}\in \left\{1,2\right\}$, i=2,4,5,6,7,8, the NEG with attacker $\xi \left(t\right)=x_{1}\left(t\right)$ and forbidden profiles set $C_{z}$ achieves robust consensus at $\eta =\delta_{2}^{1}$.

## 5. Conclusions

In this paper, we have considered the robust consensus of NEGs with attackers and forbidden profiles, and presented some new results. Based on the algebraic representation of NEGs with attackers and forbidden profiles, we have proposed a necessary and sufficient condition for the robust constrained reachability of NEGs, which is an effective tool for the robust consensus control design. In addition, by constructing a series of robust reachable sets, we have presented a constructive procedure to design state feedback controls for the robust consensus of NEGs with attackers and forbidden profiles.

It should be pointed out that one can check the robust consensus of NEGs based on the simulation from a table like Table 2. However, the simulation method may be somewhat blind. Compared with this classic method used in game theory, the STP based theoretical framework avoids the blindness of finding a suitable control strategy.

## Figures and Tables

**Table 1:** Payoff matrix of snowdrift game.

$x_{1}$\$x_{2}$	1	2
1	(3, 3)	(2, 4)
2	(4, 2)	(0, 0)

**Table 2:** Payoffs → Dynamics of the NEG.

**Profile**	**11111**	**111112**	**11121**	**11122**	**11211**	**11212**	**11221**	**11222**
$c_{1}$	6	5	5	4	8	4	4	0
$c_{2}$	6	6	8	8	5	5	4	4
$c_{3}$	6	8	6	8	5	4	5	4
$c_{4}$	6	5	6	5	6	5	6	5
$c_{5}$	6	6	5	5	6	5	5	5
$f_{1}$	1	2	2	2	2	1	1	2
$f_{2}$	1	1	2	2	2	1	1	1
$f_{3}$	1	2	1	2	2	1	1	1
**Profile**	**12111**	**12112**	**12121**	**12122**	**12211**	**12212**	**12221**	**12222**
$c_{1}$	6	5	5	4	8	4	4	0
$c_{2}$	6	6	8	8	5	5	4	4
$c_{3}$	5	4	5	4	4	0	4	0
$c_{4}$	8	4	8	4	8	4	8	4
$c_{5}$	5	5	4	4	4	5	4	4
$f_{1}$	1	1	2	2	2	1	2	2
$f_{2}$	1	1	2	2	2	1	2	2
$f_{3}$	2	1	2	2	2	2	2	1
**Profile**	**21111**	**21112**	**21121**	**21122**	**21211**	**21212**	**21221**	**21222**
$c_{1}$	6	5	5	4	8	4	4	0
$c_{2}$	5	5	4	4	4	4	0	0
$c_{3}$	6	8	6	8	5	4	5	4
$c_{4}$	5	4	5	4	5	4	5	4
$c_{5}$	8	8	4	4	8	8	4	4
$f_{1}$	1	2	1	2	2	2	1	2
$f_{2}$	2	2	1	2	2	2	2	2
$f_{3}$	1	2	1	2	2	2	1	2
**Profile**	**22111**	**22112**	**22121**	**22122**	**22211**	**22212**	**22221**	**22222**
$c_{1}$	6	5	5	4	8	4	4	0
$c_{2}$	5	5	4	4	4	4	4	0
$c_{3}$	5	4	5	4	4	0	4	0
$c_{4}$	4	0	4	0	4	0	4	0
$c_{5}$	4	4	0	0	4	4	0	0
$f_{1}$	1	1	1	1	2	2	2	2
$f_{2}$	1	1	1	2	2	1	2	2
$f_{3}$	1	1	1	2	2	2	1	2

**Table 3:** Payoff matrix of boxed pigs game.

$x_{1}$\$x_{2}$	1	2
1	(1, 5)	(−1, 9)
2	(4, 4)	(0, 0)
